# A Discussion of the Fee Problem

**Published:** 1891-08

**Authors:** William Barker


### A DISCUSSION OF THE FEE PROBLEM.[1]

BY WILLIAM BARKER, D.D.S.

PRACTITIONERS of dentistry may be likened to travellers on the high-road of ancient Thebes, to whom the Sphinx propounded her riddles.

We cannot long travel the high-road of dentistry without encountering the Sphinx, who will propound to us, not a veritable riddle, it is true, but a problem on whose satisfactory solution no small degree of our peace and success must depend. It is the fee problem. Fortunately for us, our modern Sphinx imposes no death penalty for

---

[1] Read before the Academy of Dental Science, Boston, April 12, 1891.

failure to solve the problem, and we are permitted to pass on, ever revolving, and, as yet, rarely solving the problem either to our own or the entire satisfaction of all our patients. Œdipus, we know, solved his riddle, whereupon the Sphinx destroyed herself.

We have no expectation of discovering to you a modern Œdipus, and only hope to say something which may help to place the whole question, if possible, on a more nearly scientific basis than it at present occupies, for we are persuaded that in a matter involving the reciprocal obligations of performer and receiver of professional services, there may be some way of arriving at an equitable, and possibly scientific, theory of adjustment of the ever-recurring problem of where the claims of a performer of professional service to remuneration cease, and where the obligation of the recipient of the service is discharged.

It is admitted at the outset that our problem contains factors so numerous, varied in character, and uncertain of validity, as to make a strictly scientific balancing and adjustment of them reflect itself in an equation, in any given case, not only exceedingly difficult, but perhaps impossible.

The conundrum of the street or club we may decline to consider at all, or, if found too difficult on consideration, we may give it up; but when any act or procedure in which we are participants, and of which goodness or badness, justice or injustice, may be predicated, is presented for our examination, as moral beings we may not absolutely decline its consideration because of its perplexing nature or the apparent hopelessness of arriving at a correct solution. Remembering how many problems of science, of geology, of astronomy, chemistry, or music have only been solved after many years, and as the result of the connected efforts of recurring generations of students, we may serenely proceed to the study of our theme or problem, confident that the truth, if any shall be presented, will be garnered, and the chaff of error be scattered by the winds of criticism.

If John Doe, as a farmer, ploughs, plants, and tills a field for Richard Roe, and receives remuneration therefor, he is said to be an earner of and a receiver of wages. If John Doe, as a lawyer, draws a will or prepares a brief for Richard Roe, or if, as a physician, he tends him during a fever, or if, as a dentist, he fills his tooth, removes a ranula, or constructs for him an artificial denture, and receives remuneration therefor, he is said to be a professional man, and as such, to be an earner of fees. Why this distinction without any essential difference it would be interesting to know.

The word fee, in the sense in which we here use it, as payment for service, is a word of Scotch origin, meaning cattle. Cattle were used as currency, or a medium of exchange, in the same way that peltry, shells, gold-dust, and other articles of general desire or value, have been employed by rude and primitive people at various times. Cattle, or fe, fee, fey, fie, feoh, were essentially money, as we now use the term and employ the article, and when service of any kind was rendered, it was paid for in fee or in cattle. How the term came to be restricted in its use as at present, we have been unable to ascertain. We apprehend, however, that the distinction in terms, without any essential difference in meaning at any rate, is perpetuated, and finds its chief support and value for many in the fact that in the *popular* mind a more or less hazy idea exists that somehow more honor attaches to the earning or receipt of a fee than to a wage or salary.

The term fee *is*, however, employed as descriptive of remuneration for a much larger and *less* honorable class of services than at first thought is apparent. Lawyers and doctors, horse doctors and corn doctors are paid in fees. Whoever performs the marriage ceremony, be the person a church or civic functionary, is paid in a fee. Court officials are very generally paid in fees. Pilots are paid in fees. We are said to fee the waiter or the chamber-maid, and when we conform to the porter's idea of a *real* gentleman we bestow a fee, while the stud stallion, the herd bull, the autocrat of the kennel, the monarch of the sheep-fold, and other domestic animals kept for breeding purposes are, in that capacity, said to be earners of fees for their owners.

Nor is the fee, as is quite commonly supposed, arbitrarily fixed by law or custom as payment for a definite service. John Doe, the farmer, receives wages according to the size of the field he tills or the time he works.

The fee of John Doe, the lawyer, is supposed to correspond, approximately at least, to the length or difficulty of the will or other document he draws, but it is commonly modified by the ability or willingness of his client to pay, or, what is the same thing, is gauged by the *value* of his services to his client.

What we desire more particularly to examine, in this paper, is whether the price, or fee, which is demanded for a service should be gauged by its *cost* or its value,—*i.e.*, should cost be the limit of price, or should value be the limit, or a factor even, in determining price?

The adoption of a standard of weights and measures secures not only a ready means of determining the exact relation of quantity existing between two or more portions of any material, but it *helps to secure*, as a secondary result, in the vast bulk of commercial transactions, substantial justice between buyer and seller. Poor judgment, innocence, inexperience, or even ignorance, finds in the standard a ready means of determining the exact quantities, if not the relative *values*, of commodities to be exchanged without being compelled to assume a defensive or suspicious attitude against chicane or unscrupulousness.

We say the standards of weights and measures *helps to secure justice* in commercial transactions.

A standard *based on equities* is required, however, to supplement the material standard.

Let us employ an illustration to make our meaning clear:

Eliminating needless factors and complications, let us assume Brown and Jones to be men of equal natural capacities in all directions. Equal application to any given occupation or art would result in equal efficiency in each. They are both fishermen. Brown, influenced by environment, devotes *his* time and energies to catching cod. Jones, likewise determined by his environment, catches bass. Should they desire to ascertain the relative weights of a cod and a bass, recourse would naturally be had to the scales as a standard of weight.

If we now suppose them to be catching fish for their own consumption, there being no outside market, and that neither is content with one variety of fish, and they desire to exchange with each other the catch of any day or week, what ought to be the basis on which the exchange should be made?

To use the scale now would only be to determine the relative weight of the two catches, not assisting even to determine their relative *worth*, which must be determined by a different standard.

Is it not apparent that, as between these two men, if neither is to gain any advantage over the other, if one is not to get something for nothing, while the other is to get nothing for something, the exchange must be made on the basis of cost? And supposing the amounts of cost, measured in time, wear of tackle, etc., and repugnance overcome, to be equal, one week's catch of cod ought to exchange for one week's catch of bass.

In such a case as we have been supposing, it would be an easy, and in time probably a natural, step for Brown and Jones to establish and recognize, for convenience in exchanging less than a week's or a day's catch, a relation of value compared conveniently in weight, but really measured in cost of production between the two kinds of fish, in much the same way that the relative values of gold and silver are established by law,—about one to sixteen.

At bottom, the relation is seen to be based on relative cost of production. A given amount of labor applied to the production of gold yielding one, and an equivalent amount applied to the mining of silver producing sixteen, those quantities are said to be equal in value, and exchange freely on that basis.

Cost, then, if it can be ascertained, furnishes an equitable and approximately if not a perfectly scientific measure of price.

To say that a thing is worth what it will bring, to employ the language of the market, is to say not only that value is, but that it should be, the measure of price; is to say that we may, because we can, demand from a famishing man for the measure of wheat which has cost us an hour's time and labor, and the overcoming of some slight repugnance to produce, a price equivalent to many hours or even weeks of time and labor and the overcoming of intense repugnance.

The necessities of the would-be consumer of our wheat is a factor we have not to consider, and may not take advantage of. To do so is to get something for nothing; is to appropriate as an idler, and by means of essential compulsion or duress, to our own use, the rewards of another's industry.

The ability or willingness to pay, or the necessities under which a purchaser rests, we repeat, are not factors which the seller has a right to consider or take advantage of.

The practice which many dentists more or less covertly pursue, as they say, "sizing a patient up," to estimate how heavy a fee he will stand, of course violates the equities, and places the adjusting of fees on the low plane occupied by the bully, who overrides the rights of all not bold enough or strong enough to thrash him.

These same men expect to pay no more for a pound of steak or sugar, for a book of gold, or an excavator, than would be demanded of a day laborer or a poor student for the same thing. "One price to all" is not the general commercial practice because of its convenience so much as because of its justice.

In fact, when we study the subject and familiarize our minds with the equitable harmonies likely to grow out of the general adoption of the cost principle of price, and contrast it with the injustice, the blunting of moral perceptions, the fostering of greed and avarice, and the embruiting exaltation of self at the cost of others' necessities, involved in the adoption of the principle that a thing or a service is worth what it will fetch, or that value should be the measure of price, we are convinced that, as a rule or standard for the fixing of fees, the cost principle would prove itself valuable, convenient, scientific, and satisfactory alike to all patients and operators who believe in reciprocity of service and mean to practise the virtues involved in a *quid pro quo.*

We have here indicated a *rule*, the general adoption of which by buyer and seller, between the performer and receiver of service, would tend greatly to increase the sum of human happiness, and to check, in great measure, the growth of those two classes so dangerous to modern civilization, the millionnaire and the tramp, would tend to abolish involuntary poverty, and make the fear of poverty by the industrious a shadowy dream.

A word as to the obligation imposed on believers in the cost principle to put it in practice.

At the first blush it seems to be an *inexorable law*, binding on the conscience and demanding instant obedience; as if no man unwilling to receive more benefits than he confers could continue to receive profits, or, more accurately speaking, could longer continue the practice of fleecing his patients or patrons.

This impression will vary with the varying mental and moral make-up of different individuals. It becomes a question of casuistry which each must decide for himself. "Absolve thee to thyself. Nothing is finally sacred but thine own integrity."

"In the first place, let us remember that it is impossible, in the nature of things, to apply a principle the essence of which is to regulate the terms of reciprocity where no reciprocity exists."

The equitist, who should sell on the cost principle and be compelled to buy where that principle was ignored, would soon be compelled to put up his shutters, and the campaign for justice and reciprocity would be waged, if at all, without him. Conservation of one's own energies is essential in a long race and a protracted fight. He who wars against the world single-handed, he who swims against the current alone, is likely to sink, without affecting aught save useless labor and sinful sacrifice.

The farmer living in a section of country overrun with Canada thistles, who should attempt to exterminate them from his own farm by confining his efforts to his own acres, might be expected to succeed as well as he who should attempt to inaugurate a system of fees based on the cost principle, but which none, or few others, in the community appreciated or recognized.

If we have thus far made our meaning clear, we shall perceive that the fee which may be rightfully demanded for a dental service will be, ideally, an exact equivalent for the time, labor, skill, and repugnance overcome in the direct performance of the particular operation, also the time and labor, or their equivalent, *indirectly* expended or consumed in the performance of the service while pursuing preparatory studies and acquiring the requisite qualifications to perform the service at all. If, reckoning in each and every factor legitimately contributing to the perfected service, we find it represented, say, by twenty, then that is the measure of the fee we may demand, no more no less.

The president of one of our leading universities, in a recent public address on socialism, expressed the wish that the hope of the socialists could be realized, but affirmed his disbelief in the practicability of the doctrines, because, among other reasons, as he thought, of the impossibility of arriving at the labor cost of productions.

While we admit the force of the objection and concede the impracticability of reaching in every case an exact measure of the absolute and ideal cost of dental operations, we yet contend that an honest *attempt* to do this is much more likely to secure the measure of an equitable fee than is the too prevalent method of sizing a patient up by the clothes he wears, the house he lives in, or the equipage he drives.

We contend, *at least*, for a reasonable correspondence between cost and price.

Criticism will converge upon the word reasonable. What is to be considered reasonable?

An excavator twelve inches long all would agree was too long to be convenient, while one only three inches long most of us would think too short to be useful; and while as to the length of his excavators as well as to the amount of his fees each dentist will and should be his own judge, yet in determining the amount of his fees he should be governed by other considerations than his own narrow interests or desires. It must be borne in mind that cost is the rightful limit of price.

We are of the opinion that twenty dollars per hour violates a reasonable correspondence between cost and price in one direction, and that two dollars per hour violates it in the other direction; fifteen dollars per hour, as a constant price, we are inclined to opine still violates the reasonable correspondence we are contending for, while ten dollars per hour as a constant price comes in our opinion perilously near it. "Let every man be fully persuaded in his own mind."

We think the time basis of fees, as a rule, can be made to more nearly harmonize with the cost principle of price than a system based arbitrarily on minimums, or the size of cavities, or the materials employed, or all combined.

We believe it to be inequitable, as well as unbusiness-like, to tacitly or openly and avowedly perform any beyond the most trivial service without a fee, with the covert or open intention of "getting square with them" at some other sitting, or, as we have sometimes heard, of getting it out of somebody else. Let every tub stand on its own bottom.

If we extract teeth and insert artificial dentures, let each be the subject of a fee, as much as would be the case if we amputated a leg and supplied a cork substitute, or dug and walled a cellar and afterwards erected a house over it. The busy dentist unquestionably should receive a fee for broken appointments, for a reason so obvious it need not be indicated. Fees for services to children, if conformable to the cost principle, should at least be as large as for adults, because of the ordinarily greater degree of repugnance overcome in serving them.

Conformity to the cost principle would very generally considerably increase the fees for work on devitalized teeth and for cleansing. The disproportion between cost and price for services in regulating, to the average dentist, has always been cause for legitimate complaint.

But our object is not to discuss specific cases, but to unfold a principle by reference to which cases may be adjusted as they arrive.

One thought more. The exaction of too high fees, not only drives patients away from the practitioner exacting them, but not infrequently influences them to forego the benefits of dentistry to a harmful extent. Flying from the competent and honest operator, but dishonest fee-maker, the tendency is towards the quack operator and quack fee-maker. Scylla on the one hand, Charybdis on the other, as they make the course. Many, it is true, wisely steer the middle course, and are faithfully served as to operations and honorably dealt with as to fees.

A reasonable correspondence between cost and fees, if not in every case swelling the purse as would a fee violating that correspondence, on the high side, will yet, to the equitist at least, yield reasonable satisfaction and greatly tend to enlarge the field over which the benefits of dentistry shall be enjoyed.

